# Advances in Multi-Modal Biomarkers for Immunotherapy Response in Non-Small Cell Lung Cancer: ctDNA, Microbiome, and Radiomics

**DOI:** 10.3390/cancers18081281

**Published:** 2026-04-17

**Authors:** Turja Chakrabarti, Matthew Lee

**Affiliations:** City of Hope Comprehensive Cancer Center, 1500 East Duarte Road, Duarte, CA 91010, USA; tchakrabarti@coh.org

**Keywords:** lung cancer, immunotherapy, artificial intelligence, circulating tumor DNA, microbiome, radiomics

## Abstract

Lung cancer remains the leading cause of cancer-related mortality worldwide, and although immunotherapy has transformed the treatment landscape of advanced non-small cell lung cancer (NSCLC), durable benefit is limited to a subset of patients. PD-L1 immunohistochemistry and tumor mutational burden, while clinically utilized, demonstrate imperfect predictive capacity, underscoring the need for more robust biomarkers. This review highlights emerging multimodal biomarkers—including circulating tumor DNA (ctDNA), the gut microbiome, and artificial intelligence (AI)-driven radiomics—as promising tools to enhance the prediction of immunotherapy response. Longitudinal ctDNA monitoring offers a minimally invasive method to assess tumor burden dynamics, detect early molecular response, distinguish pseudo-progression from true progression, and stratify risk, with ctDNA clearance correlating with improved survival outcomes. The gut microbiome has also been associated with ICI efficacy, as specific bacterial taxa and composite scoring systems correlate with treatment response, though methodological heterogeneity limits clinical translation. Radiomic analyses leveraging CT and PET imaging extract quantitative tumor features that, when integrated with clinical and molecular data, demonstrate improved predictive performance compared to single-modality approaches. Despite promising advances, challenges including assay standardization, external validation, data harmonization, interpretability of AI models, and infrastructure requirements remain barriers to widespread adoption. Multimodal integration of genomic, microbiome, and imaging biomarkers represents a critical step toward precision immuno-oncology, with prospective validation needed to translate these approaches into improved outcomes for patients with advanced NSCLC.

## Simple Summary

Lung cancer is the leading cause of cancer deaths worldwide. Immunotherapy helps treat this cancer, but it only works well for some patients. Scientists are exploring new ways to predict who will benefit, including blood tests, gut bacteria, and AI imaging. These methods can monitor tumor changes and predict treatment success better than current tests. However, there are still challenges to make these tools routine in clinics. Combining these different biomarkers could improve personalized treatment for lung cancer in the future, helping more patients receive effective care. We review the use of DNA blood tests, gut microbiome data, and AI imaging platforms in lung cancer treatment decisions. We also propose an AI model that can combine multiple types of data to provide more accurate treatment predictions and recommendations for patients with lung cancer.

## 1. Introduction

Lung cancer remains the most lethal malignancy globally, accounting for nearly 18% of all cancer-related mortality [1]. The development of immune checkpoint inhibitors (ICIs) targeting the programmed death ligand-1 (PD-L1) pathway has revolutionized the treatment landscape in lung cancer. Despite decades of advances in screening, diagnosis, and ICI treatment paradigms, patients with metastatic lung cancer have poor outcomes, and long-term survival beyond five years remains below 10%. Clinical practice relies heavily on PD-L1 immunohistochemistry (IHC) assessment, with the tumor proportion score (TPS) guiding most immunotherapy decisions. For instance, biomarker-driven patient selection gained widespread clinical adoption as the Keynote-024 trial demonstrated the superiority of pembrolizumab monotherapy over chemotherapy in patients with PD-L1 TPS ≥ 50% [2]. Of note, not all patients expressing high PD-L1 levels respond to checkpoint blockade, as objective responses remain 40–50% even within this biomarker-selected population. As such, PD-L1 expression alone is insufficient to predict ICI response [3]. Similarly, tumor mutational burden (TMB) has shown limited and inconsistent predictive capacity in immunotherapy response [4]. Future directions in lung cancer treatment involve a shift toward multidisciplinary, biomarker-driven, precision oncology approaches to improve patient outcomes.

Current biomarkers are severely limited in assessing the inherent complexity of tumor–immune interactions, a complexity that cannot be adequately captured by a single molecular assay. The tumor microenvironment (TME) is a dynamic ecosystem of malignant cells, surrounding immune cells, dense stromal tissue, and often harsh immune-inhibitory signaling. PD-L1 expression represents only one dimension of this multifactorial biology and does not account for the dynamic status of tumor-infiltrating lymphocytes, immunosuppressive cell populations, or the spatial characteristics of the TME. Moreover, evidence indicates that TME is influenced by the composition of the human microbiome.

Future directions in precision oncology will inevitably recognize that immunotherapy response is governed by multiple interacting biological systems, which prompts a shift towards integrated biomarkers. High-throughput sequencing, computational biology, and artificial intelligence have unraveled unique opportunities to develop novel integrated biomarker platforms that are more precise and accurate in clinical oncology prediction. A systems-level perspective demonstrates that predictive accuracy improves when complementary data streams are combined and analyzed synergistically.

This review will provide a comprehensive review of predictive and prognostic biomarkers for immunotherapy response in advanced non-small cell lung cancer (NSCLC), with a focus on circulating tumor DNA (ctDNA), the microbiome, and artificial intelligence (AI) driven radiomics. This review aims to reflect on the current consensus and emerging evidence, integrating findings from recent meta-analyses, aggregate clinical trial data, and translational studies. We also review lessons learned from the COVID-19 pandemic in advancing clinical AI utilization, especially in radiomics. Finally, we provide a proposed architectural framework for a multimodal AI pipeline.

## 2. Circulating Tumor DNA in Advanced NSCLC Treated with Immunotherapy

Liquid biopsies utilize circulating tumor DNA (ctDNA) as a minimally invasive, dynamic biomarker for the real-time monitoring of tumor burden and molecular response. Mechanistically, cancer cells release cell-free DNA into the bloodstream, which is then captured by PCR or targeted sequencing methods. Longitudinal monitoring of ctDNA assays enables the detection of ICI therapy responders before imaging changes [5]. A meta-analysis of 3047 NSCLC patients receiving systemic therapies, including ICI and targeted therapy, found ctDNA reduction associated with significant improvements in overall survival (OS) and progression-free survival (PFS) [6]. Obtaining baseline ctDNA levels and monitoring for changes during treatment may help identify patients who could benefit from treatment escalation or de-escalation. Liquid biopsy methods can also identify specific prognostic and predictive biomarkers, such as KRAS or TP53, highlighting treatment response and potential mechanisms of resistance [7]. Overall, ctDNA offers a personalized tool in precision oncology to help predict response, adjust treatment, and evaluate the impact of immunotherapy [5,6,7,8,9,10,11,12,13].

Clinical data demonstrate the immense utility of ctDNA in clinical decision-making. For instance, patients whose ctDNA levels drop sharply soon after initiation of ICI are more likely to experience longer periods without disease progression and trend towards improved overall survival [7,10,14,15,16,17]. Such findings highlight liquid biopsies as a unique biomarker in monitoring treatment response and predicting clinical outcomes, thereby optimizing immunotherapy. Moreover, an aggregate analysis of eight clinical trials (n = 940) confirmed that ctDNA clearance within 10 weeks of ICI therapy initiation was independently associated with improved PFS and OS, after adjusting for clinical covariates and radiographic response [18].

Recent clinical studies have shown that changes in ctDNA levels can outperform radiologic analysis of response to immunotherapy [2,5]. A machine learning algorithm modeled liquid biopsy metrics in the phase 3 IMPower 150 clinical trial and found that ctDNA changes preceded changes in tumor size on computed tomography (CT) imaging and more accurately predicted outcomes [6]. Accordingly, ctDNA can be utilized for risk stratification in patients receiving immunotherapy. Notably, a reduction in ctDNA can help distinguish pseudo-progression, a phenomenon unique to immunotherapy, characterized by immune cell infiltration that may appear as an enlarging tumor, rather than true tumor progression [8,10]. As such, ctDNA monitoring offers clinicians a more accurate means of monitoring treatment response.

Despite the promise of ctDNA as a predictive and prognostic biomarker for immunotherapy, several limitations hinder its widespread clinical application in lung cancer. A significant challenge arises from technical variability across ctDNA assays, which can lead to different results depending on the methods used (such as if they are tumor informed-requires tissues or tumor agnostic-does not require tissue) each with their own various sensitivities and specificities [19]. At present, there is a lack of standardized thresholds for interpreting ctDNA levels, creating challenges in comparing results across studies or institutions. There remains a critical need to harmonize ctDNA testing platforms to enable reliable, reproducible results in clinical settings. In addition, ctDNA assays have decreased sensitivity in patients with low tumor burden [20]. Several variables prior to assay execution, such as sample collection, processing, and storage, can affect ctDNA results. Such factors must be carefully controlled to allow for accurate and reliable liquid biopsy results.

In the context of lung cancer, ctDNA monitoring for immunotherapy remains mostly investigational. However, studies show promise for ctDNA analysis in targeted therapy for EGFR-mutated lung cancer. A post hoc analysis of patients receiving adjuvant osimertinib in the ADAURA trial found that tumor-informed ctDNA-based molecular residual disease (MRD) testing could identify patients who might benefit from extended adjuvant osimertinib [21]. Efforts are underway to define standardized ctDNA cutoffs and incorporate liquid biopsies into adaptive clinical trials. Future goals include making ctDNA an early surrogate endpoint in trials and clinical decision-making. A summary of studies and trials that have evaluated ctDNA in advanced NSCLC are included in Table 1.

## 3. The Microbiome as a Predictive and Prognostic Biomarker in NSCLC Immunotherapy

The diversity and composition of the gut microbiome are closely associated with the efficacy of ICI therapy in patients with advanced NSCLC. The study of gut microbiota and dietary patterns in patients receiving ICI found associations between microbiome composition and clinical outcomes [22,23,24]. Of note, one study analyzed patient stool samples to identify specific taxa (e.g., Ruminococcaceae, Akkermansia, Alistipes, Eubacterium ventriosum) linked to favorable outcomes, while others (e.g., Rothia, Streptococcus salivarius) are associated with resistance or a poor prognosis [23]. Microbiome composition can also be analyzed using machine learning indices, such as the TOPOSCORE, or PCR-based assays to risk-stratify patients with advanced NSCLC and predict response to immunotherapy [24,25]. Specifically, the TOPOSCORE is a diagnostic scoring system to evaluate gut dysbiosis based on the balance between two interacting groups of bacteria: 37 associated with poor responses to ICIs vs 45 associated with favorable responses to ICIs [25]. Beyond the microbiome, several modifiable factors, such as proton pump inhibitor use, probiotic use, and dietary choices, alter the gut environment and may impact ICI treatment outcomes [22].

While findings linking gut microbiome composition to immunotherapy outcomes have been promising, several challenges limit the translation of these discoveries into validated clinical applications. Notably, there is a lack of standardization in protocols for sampling and analyzing the microbiome [26]. Variability in microbiome assessment methods makes it difficult to compare results across studies and tumor types and establish standardization benchmarks. Moreover, prospective validation is required to confirm the clinical significance of microbiome-based biomarkers.

## 4. Artificial Intelligence-Based Radiomic Analysis

Radiomics refers to the data-driven analysis of quantitative features derived from CT images, such as texture, shape descriptors, and intensity statistics [27,28]. By deploying artificial intelligence and deep learning methods in radiomic analysis, predictive insights into immunotherapy clinical outcomes can be gained. Radiomic features have been associated with molecular characteristics, such as genomic/mutational status such as EGFR, histological subtype, and treatment response [29]. Accordingly, radiomics studies can non-invasively predict ICI response and clinical outcomes in patients with advanced NSCLC [30]. A retrospective analysis of 385 NSCLC patients treated with ICIs extracted radiomic features from pretreatment CT scans to build a predictive model for PD-L1 status and PFS, achieving an area under the receiver operating characteristic curve (AUROC) of 0.63 [31]. Another retrospective analysis of 296 NSCLC patients developed a COMB-Radscore from PET and CT images that achieved greater predictive performance with AUROC of 0.819 [32].

Current limitations in radiomics include technical heterogeneity in imaging acquisition protocols, lack of standardization, and limited external validation. Additionally, most studies are retrospective, and regulatory approval and clinical implementation are still pending. Future initiatives must address these unique challenges. Prospective validation, harmonization of radiomic and AI-driven computational pipelines, and the integration of these technologies into clinical workflows are essential steps to ensure consistency, reliability, and seamless adoption in healthcare settings.

### 4.1. Graph Neural Networks for Spatial Modeling

Graph Neural Networks (GNNs) demonstrate an evolution in radiomic analysis by modeling spatial relationships beyond traditional approaches [33]. GNNs in radiology represent medical images as graphs, with regions of interest corresponding to nodes and spatial proximity encoded as edges. Lung cancer imaging is well-suited for the GNN representation approach as it addresses the complexities of tumor heterogeneity, non-solid ground glass patterns, lymphangitic spread, and tumor–microenvironment interactions [34]. In the context of early-stage lung cancer, graphs were derived from segmented CT regions and captured spatial relationships via graph convolution, yielding survival predictions superior to traditional staging, machine learning, and tumor-focused convolutional neural networks (CNNs) [35]. The application of GNNs to lung CT scan segmentation can effectively capture complex tumor heterogeneity. Integrating GNN models with radiomics encoders achieved improved segmentation accuracy when compared to conventional methods [34]. Accordingly, GNNs could be applied to model the spatial distribution of radiomic features across tumor regions, thereby detecting imaging heterogeneity patterns associated with treatment resistance or response.

### 4.2. Vision Transformers

Vision transformers (ViT) have the potential to transform medical imaging analysis through their ability to capture global context. ViTs deploy self-attention mechanisms that allow each image patch to learn from all other patches, thereby capturing tumor heterogeneity across lung fields [36,37,38]. Fundamentally, ViT architecture involves inputting images into non-overlapping patches, creating embeddings, and processing through multiple transformer encoders [38]. Uniquely, Radiomics-Embedded Vision Transformer (RE-ViT) represents an integrated framework that combines handcrafted radiomic features with patch-wise ViT embeddings to enhance medical image classification across diverse datasets [39]. ViTs offer several key capabilities for NSCLC immunotherapy classification by modeling tumor heterogeneity, revealing global context, and enabling clinical interpretability through map visualization, which clarifies which image regions contribute to predictions.

### 4.3. Lessons from the COVID-19 Pandemic

The COVID-19 pandemic sparked advances in AI based lung imaging analysis, which created the infrastructure, data sets, and methodological expertise directly applicable to lung cancer research [40,41]. The urgent need for rapid, accurate COVID-19 diagnosis drove the development of deep learning models for chest CT interpretation, resulting in large annotated datasets, validated algorithms, and increased clinical acceptance of AI and pulmonary imaging workflows.

Multiple deep learning architectures were rapidly developed and validated for COVID-19 detection on chest CT. EfficientNet-B5, a CNN architecture, achieved 97.6% accuracy in classifying COVID-19 positivity [40]. ResNet-based models achieved individual patient-level accuracy of 95.2% and per-image accuracy of 98.9% for COVID-19 pneumonia detection, demonstrating comparable performance to expert radiologists while reducing reading time by 65% [42]. Global collaborative efforts developed deep learning algorithms trained on diverse cohorts comprising over 4500 patients, achieving 93% specificity in detecting COVID-19 pneumonia [43].

A systematic review and meta-analysis of COVID-19 CT deep learning studies revealed that ResNet architectures demonstrated the best diagnostic performance [44]. Such foundational work established best practices for lung CT imaging analysis, including data augmentation strategies, transfer learning approaches, and interpretability methods, such as weighted class mapping to visualize model attention on ground-glass opacities and consolidations [40,44,45].

The COVID-19 experience offers several crucial lessons for predicting lung cancer immunotherapy response. The pandemic demonstrated the feasibility of rapid multi-institutional data sharing and collaborative model development. Furthermore, pre-trained models developed on COVID-19 datasets represent valuable starting points for transfer learning in lung cancer applications. Additionally, the pandemic accelerated clinical acceptance of AI-assisted radiology tools.

In a landmark overview of healthcare data analytics, Fei et al. comprehensively synthesize how the pandemic served as a forcing function for machine learning tools, statistical inference models, and distributed computing systems to address urgent clinical problems at scale [46]. Critically, this work highlighted the domains catalyzed by the pandemic, namely: CNNs, recurrent neural networks, GNNs, and high-dimensional statistical inference for genomic biomarker discovery. These are precisely the architectures now being deployed for multimodal NSCLC immunotherapy prediction.

Beyond imaging, the pandemic ushered in the use of GNNs for computational epidemiology problems, specifically encoding the structured network features into architectures for infection source detection and contact tracing [47]. Successful implementation of GNNs enabled accurate identification of superspreading infection clusters in Singapore and Taiwan, using data from the SARS-CoV-2 2003 and COVID-19 pandemics [47]. As such, these use cases provided the framework to apply GNNs to model tumor heterogeneity in lung cancer [34,35].

### 4.4. Multimodal Transformer Architecture

Developing technical architectures that can integrate genomic, imaging and clinical data through cross attention mechanisms, offer a transformative opportunity for AI driven clinical decision support. Multimodal transformers deploy separate analytic encoders for each data modality, followed by cross-discipline fusion modules that learn interactions between modalities [48,49]. In relation to lung cancer immunotherapy prediction, multimodal transformers have the potential to integrate CT/PET radiomics with ctDNA genomic features and clinical variables. Foundation models pretrained on large-scale pathology images and text can effectively combine complementary information from images and clinical reports to improve outcome prediction, including immunotherapy response in lung cancer [50]. Deep learning frameworks that incorporate adversarial training have consistently improved cancer survival prediction by generating modality- and variant-specific representations [51].

Attention-based multimodal fusion transformers predict treatment response with AUCs over 0.80 by modeling hierarchical interactions among imaging, molecular, and clinical data. Specifically, hierarchical collation transformers that characterize interactions between digital pathology-based visual mobilization and radiologic features demonstrated improved performance over unimodal approaches in survival prediction [52]. Overall, these architectures promote clinical decision support by providing interpretability through attention weights that reveal which imaging regions and molecular features contributes most to predictions [53,54].

### 4.5. Clinical Validation of Multimodal AI Models

The translation of multimodal AI biomarkers from concept to clinical validation represents a key step toward clinical adoption. A multicenter effort developed the Lung Cancer Neo-adjuvant Immunotherapy–Chemotherapy Response Predictor (LC-NICER) system (NCT06285058). This system integrates longitudinal radiomics, deep learning microenvironmental context, and habitat imaging of tumor subregional dynamics to predict pathologic response. LC-NICER demonstrated 10% absolute accuracy improvement over PD-L1 testing and 18% over RECIST 1.1 criteria [55].

The DEEP-Lung IV study (NCT04994795) is a real-world multicentric observational study including over 4000 patients with NSCLC treated with first-line immunotherapy [56]. This study aims to validate a multimodal deep learning algorithm that integrates clinical, pathological, and baseline CT scan radiomics data to predict individual patient response to immunotherapy. A proof-of-concept analysis in 63 patients demonstrated that a logistic regression algorithm achieved AUC of 0.85 for predicting response at first evaluation [56]. Notably, the prediction represented features of all data modalities contributing to model performance. Studies exploring AI and deep learning approaches to predicting immunotherapy are summarized in Table 2.

## 5. Multimodal Biomarker Integration and Future Perspectives

Emerging studies have shown that a multimodal integration of ctDNA, and AI/radiomics can demonstrate superior predictive performance for immunotherapy benefit in NSCLC compared to single biomarkers, though significant challenges in standardization, validation, and clinical implementation remain.

In terms of ctDNA, an example of the combination of ctDNA and AI was examined in a study that utilized a machine learning model (XGBoost) that incorporated 25 prognostic genomic features from pre-treatment ctDNA profiling [57]. The model was able to achieve a robust discrimination (AUC 0.82 training, 0.79 validation, 0.77 test) for predicting PFS in NSCLC patients receiving ICIs along with also identifying TP53, BRCA2 and NOTCH1 as predictive biomarkers [57]. Of note, XGBoost is distinct from deep learning models in that it is a gradient boosting framework that combines weak decision trees into a strong predictive model through iterative optimization.

Deep learning models that integrate clinical and genomic data, histology radiomics, and deep features demonstrate superior performance (AUC 0.80–0.90) compared to unimodal approaches [58,59,60]. Similarly, a multi-dimensional model combining clinical features (modified lung immune predictive index), radiomics score, and immune signatures showed better predictive efficacy (C-index 0.721 for PFS, 0.727 for OS) than individual components [61]. A multiomic graph and model that combines radiomics, pathological biomarkers (PD-L1, STK11, KRAS), and clinical variables achieved C-index 0.71 (95% CI 0.61–0.72) for PFS prediction, outperforming combination clinical models (C-index 0.68) and clinical-only models (C-index 0.58) [62]. Combined models incorporating inflammatory indices (NLR, PLR), PD-L1 expression, and molecular alterations identified distinct patient clusters with significantly different outcomes (ORR ranging from 7% to 41%) [63]. All these examples show how different factors can be integrated into a unified model in order to help predict various clinical outcomes more accurately.

However, despite these advancements in prediction models there are limitations in this approach. Variations in image acquisition parameters, CT reconstruction kernels, slice thicknesses, and device manufacturers can create significant challenges for radiomics model generalizability [64]. Without image harmonization, models that perform well in discovery cohorts (AUC 0.69) often fail external validation (AUC 0.52–0.57) [64]. Moreover, most AI and multimodal models demonstrate excellent single-institution performance but fail to maintain accuracy in external cohorts—the “validation gap” [65]. Of 90 AI studies for ICI prediction reviewed, none provided high-level evidence for immediate practice change, and no prospective trials incorporated AI methodologies from the outset [66]. The lack of large, annotated, multi-institutional datasets limit robust model development and validation [66,67]. Moreover, complex deep learning models often function as “black boxes,” making it difficult for clinicians to understand predictions and trust clinical recommendations [65,68].

Currently, a multi-omic approach requires specialized infrastructure, including high-throughput sequencing platforms, bioinformatics pipelines, and computational resources that are not universally available and have yet to be integrated [67,68]. Ongoing translational research focuses on prospective validation of multimodal models in randomized trials, development of standardized data collection and analysis pipelines, and creation of interpretable AI frameworks that provide actionable clinical insights [66,67].

### 5.1. Framework for Multimodal AI Architecture

The integration of ctDNA, gut microbiome profiles, and AI driven radiomics represents a unique opportunity to advance precision oncology in lung cancer. We propose a comprehensive evidence-based technical architecture that addresses the unique challenges of fusing heterogeneous biomedical data to produce clinically interpretable predictions.

### 5.2. Architectural Overview

The proposed design employs a multimodal fusion strategy that processes each data modality with specialized encoders, followed by integration via cross-modality attention mechanisms [69,70,71]. For ctDNA genomic features, we can use Fragle, a deep learning model for ctDNA quantification that analyzes the density distribution of cell-free DNA fragment lengths to estimate tumor fraction [72]. Fragle uses a neural network to learn the subtle differences in DNA fragment length distributions between tumor-derived and normal cfDNA. Fragle was found to be superior to a tumor gene panel for minimal residual disease prediction and risk stratification. Additionally, MRD-EDGE uses deep learning to enrich for single-nucleotide variants (SNVs) and detect CNVs. For lung cancer, MRD-EDGE was used to track changes in tumor fraction in response to neoadjuvant immunotherapy through plasma only disease monitoring [73].

Furthermore, Fei and colleagues described high-dimensional inference (HDI) methods, including split and smooth approaches for identifying significant predictors among thousands of genomic features [46]. These methods have direct translational applications in our proposed architecture, enabling ctDNA biomarker discovery and the identification of predictive molecular signatures in NSCLC patients receiving immunotherapy.

For imaging, we would use RE-ViT architecture to integrate radiomic features (texture, shape, intensity, statistics) with patch-level embeddings learned by the transformer encoder. Baseline CT images would be divided into non-overlapping 16 × 16 pixel patches and then projected into 512-dimensional embeddings, followed by processing through 12 transformer encoder layers [39]. In addition, we can use robust transfer learning pipelines in which models pre-trained on large COVID-19 lung imaging datasets can be fine-tuned for lung cancer imaging analysis [74]. Moreover, large-scale screening for COVID-19 catalyzed widespread CT chest usage which inadvertently identified early-stage cancers. For instance, a French database from pandemic lockdowns includes 24,390 patients with at least one CT scan and a 0.30% incidence of lung cancer, providing a rich dataset of lung imaging for AI model training [75].

Microbiome data integration will be uniquely challenging due to its heterogeneous compositional nature and high dimensionality. MicroAIbiome is an AI pipeline designed to classify cancer type using genus-level microbiome components [76]. This pipeline incorporates zero-placement, centered log-ratio transformation, and recursive feature elimination to enable robust learning from compositional microbiome data [76].

### 5.3. Fusion Mechanisms

Diverse modalities represent a critical challenge in the development of multimodal AI pipelines. To address this, we propose developing a cross-modal fusion module which allows for each data type to learn from other modalities. The fusion architecture will consist of three branches: (1) a self-attention branch where each modality attends to itself to capture intra-modal relationships; (2) a cross-attention branch where modalities attend to each other to learn inter-modal dependencies; and (3) a factorized bilinear pooling module that captures second-order interactions between modalities while maintaining computational efficiency [69,77].

### 5.4. Interpretability and Clinical Decision Support

To ensure clinical utility, the architecture must support multiple mechanisms of explanation. Shapley Additive exPlanation (SHAP) can quantify the contribution of each feature to individual predictions, identifying which ctDNA mutations, radiographic patterns, or bacterial taxa drive immunotherapy response for patients [78]. In addition, attention-based heatmaps can visualize which image regions, genomic alterations, and clinical variables receive the highest attention weights, providing clinicians with interpretable evidence supporting predictions [59,70]. For radiomics specifically, we can generate gradient weighted class activation maps (Grad-CAM) that highlight tumor regions most likely to predict immunotherapy response [79]. Additionally, for microbiome features, we can provide pathway enrichment analyses that link bacterial taxa to known immunomodulatory mechanisms [80]. This multilevel interpretability approach can help translate complex computational predictions into understandable clinical decision support.

A summary of different studies that have integrated a multimodal biomarker approach in NSCLC is presented in Table 3.

## 6. Conclusions

Although, immunotherapy has transformed the field of NSCLC management and improved clinical outcomes, there remains a need for improvement in diagnostic surveillance and biomarker utility. Several advances, such as in ctDNA, are quickly becoming increasingly predictive and prognostic biomarkers, but with limitations that can be overcome by combining with other biomarkers. These novel emerging biomarkers of the microbiome, along with the use of AI, will continue to push the field forward, but despite this promise, several key considerations must be addressed before widespread clinical implementation. Large, well-annotated datasets with standardized sample collection, sequencing platforms, imaging protocols, and clinical endpoints are essential to ensure model robustness and generalizability.

The three biomarker domains examined in this review—ctDNA, the gut microbiome, and AI driven radiomics—each offer distinct perspectives into tumor biology and immune response. Liquid biopsies offer a minimally invasive method of analyzing dynamic tumor genomic changes in real time. Meanwhile, the gut microbiome reflects the systemic immunological characteristics that determine the host immune response to checkpoint blockade. Microbiome data offers insights into personalized variability that cannot be detected by tumor studies alone. Radiomic analysis extracts quantitative features from imaging to transform clinical imaging into rich descriptive data that correlate with clinical outcomes.

Multimodal integration represents a paradigm shift in biomarker development, converging these data streams to enable unified, more precise predictive analytics. We review how predictive accuracy increasingly depends on the synthesis of complementary data, each capturing a distinct aspect of cancer biology. We reviewed architectural frameworks—Graph Neural Networks for spatial modeling, Vision Transformers for global context, and cross-modal attention mechanisms for data fusion—to provide a technical foundation required for this integrative vision. In a review of the existing literature, we found multiple AI pipelines that combine clinical, genomic, and radiomic features and demonstrate superior predictive performance compared to unimodal approaches.

Nevertheless, transitioning from research findings to validated clinical tools remains challenging. Each biomarker domain presents technical challenges: ctDNA assays require standardized platforms; microbiome assays currently exhibit methodological differences; radiomic models are vulnerable to divergent image acquisition methods. The integration of these modalities introduces further complexity as harmonizing diverse data types demands sophisticated computational approaches.

The successful translation of multimodal biomarkers will require addressing infrastructural, regulatory, and ethical issues. Bioinformatics expertise and immense computational resources required to develop and implement these diagnostic pipelines are not uniformly available across healthcare systems. This raises concerns regarding equitable access to precision oncology tools. Regulatory frameworks for AI-based clinical decision support are still evolving, and data privacy considerations will be a key tenet of future applications. Reimbursement pathways for novel integrated biomarkers and AI must be developed to enable sustainable implementation.

The lessons learned from the COVID-19 pandemic offer resources for advancing AI platform development. The rapid deployment of AI models for imaging analysis during the pandemic illustrated how the scientific community can mobilize when clinical need is urgent, and collaboration is prioritized. The tools and infrastructure created during this period, including large annotated datasets, validated deep learning architectures, and increased acceptance of clinical AI tools, have provided a foundation upon which oncology-focused applications can be built.

Looking forward, several opportunities emerge for this field. Firstly, prospective clinical trials must incorporate multimodal biomarkers from the start to generate high quality reproducible evidence needed for clinical adoption. Adaptive trial designs that utilize ctDNA dynamics, microbiome data, and integrated predictive scores to guide clinical care represent promising efforts to demonstrate clinical utility. Second, collecting data in a harmonized and standardized method will be crucial to develop effective computational pipelines that are reproducible. Moreover, the development of interpretable AI methods that provide actionable clinical insights will be essential for clinical implementation and facilitate regulatory approval.

The interpretability of complex AI-driven models remains critical for clinician trust and regulatory approval, particularly when decisions impact treatment selection. Additionally, regulatory, logistical, and ethical challenges—including data privacy, cross-institutional data sharing, assay harmonization, reimbursement, and equitable access—must be carefully navigated. As multi-omic and imaging-based decision support tools evolve, rigorous validation and thoughtful integration into clinical workflows will be necessary to translate their potential into improved outcomes for patients with NSCLC.

## Figures and Tables

**Table 1:** Summary of Key ctDNA Studies in Advanced NSCLC.

Study/Reference	Study Design	Population (N)	ctDNA Assay/Method	Key Findings	Clinical Implications
Leite da Silva et al. (2025), Ref. [6]	Systematic review and meta-analysis	3047 NSCLC patients (ICI and targeted therapy)	Multiple platforms (PCR, targeted sequencing)	ctDNA reduction associated with significant improvements in OS and PFS	Supports baseline ctDNA monitoring and longitudinal tracking for treatment response assessment
Assaf et al. (2023), Ref. [9]	Longitudinal modeling (IMpower 150 trial)	Phase 3 clinical trial cohort	Machine learning algorithm on liquid biopsy metrics	ctDNA changes preceded CT imaging changes and more accurately predicted outcomes	ctDNA may outperform radiologic response assessment; supports early molecular response monitoring
Andrews et al. (2025), Ref. [18]	Aggregate analysis of 8 clinical trials	940 patients	Multiple ctDNA platforms	ctDNA clearance within 10 weeks of ICI initiation independently associated with improved PFS and OS	Early ctDNA clearance as a surrogate endpoint; supports risk stratification within 10 weeks of therapy
Pellini et al. (2023), Ref. [14]	Prospective monitoring	Advanced NSCLC on chemo-immunotherapy	Longitudinal ctDNA monitoring	Sharp ctDNA decline after ICI initiation correlated with longer PFS and improved OS	ctDNA dynamics enable early identification of responders and risk stratification
Goldberg et al. (2018), Ref. [15]	Prospective cohort	Advanced NSCLC on immunotherapy	Targeted sequencing	Early ctDNA assessment predicted immunotherapy response before imaging	Supports incorporation of early ctDNA assessment into clinical decision-making
Herbst et al. (2025), Ref. [21]	Post hoc analysis (ADAURA trial)	Resected EGFR-mutated stage IB-IIIA NSCLC	Tumor-informed ctDNA-based MRD testing	MRD testing identified patients benefiting from extended adjuvant osimertinib	Demonstrates ctDNA utility in adjuvant setting for EGFR-mutated NSCLC
Gelmini et al. (2025), Ref. [7]	Prospective biomarker study (CORELAB)	NSCLC patients on immunotherapy	Cell-free DNA profiling	Identified specific prognostic biomarkers (e.g., KRAS, TP53) from liquid biopsy	Liquid biopsy can identify resistance mechanisms and guide targeted therapy selection

**Table 2:** AI-Based Radiomic and Deep Learning Approaches for Predicting Immunotherapy Response in NSCLC.

Approach/Architecture	Study/Reference	Data Modality	Population	Performance Metric	Key Advantages	Limitations
Traditional Radiomics (Handcrafted features)	Yolchuyeva et al. (2023), Ref. [31]	Pretreatment CT scans	385 NSCLC patients treated with ICIs	AUROC 0.63 for PD-L1 status and PFS prediction	Non-invasive; utilizes existing imaging data; reproducible feature extraction	Moderate predictive performance; sensitive to imaging acquisition parameters
PET/CT Radiomics (COMB-Radscore)	Lin et al. (2025), Ref. [32]	PET and CT images (intratumoral + peritumoral)	296 NSCLC patients	AUROC 0.819	Captures metabolic and morphologic tumor features; improved performance over CT alone	Requires PET imaging; limited external validation
Graph Neural Networks (GNNs)	Faizi et al. (2025), Ref. [34]	Segmented CT regions	Early-stage and advanced lung cancer	Superior survival prediction vs. traditional staging and CNNs	Models spatial tumor heterogeneity; captures lymphangitic spread and TME interactions	Computationally intensive; requires segmented imaging data; limited prospective validation
Vision Transformers (ViT/RE-ViT)	Refs. [36,37,38,39]	CT images (patch-based)	NSCLC cohorts	Enhanced classification across diverse datasets	Captures global context via self-attention; interpretable attention maps; models full lung fields	Large training data requirements; computational cost; limited oncology-specific validation
COVID-19 Transfer Learning (EfficientNet-B5, ResNet)	Refs. [40,41,42,43,44,45]	Chest CT	>4500 patients (COVID-19 cohorts)	97.6% accuracy (COVID-19); 93% specificity	Pre-trained models transferable to lung cancer; established best practices for CT analysis	Domain shift between COVID-19 and cancer; requires fine-tuning for oncology applications
Multimodal Fusion Transformer	Refs. [48,49,50,51,52,53,54]	CT/PET radiomics + genomics + clinical data	Multiple NSCLC cohorts	AUC >0.80 for treatment response prediction	Cross-modal attention learns inter-modality interactions; interpretable attention weights	Data harmonization challenges; requires multi-modal data availability
LC-NICER System	NCT06285058, Ref. [55]	Longitudinal radiomics + deep learning TME context + habitat imaging	Multicenter NSCLC cohort	10% improvement over PD-L1; 18% over RECIST 1.1	Integrates tumor subregional dynamics; prospective multicenter validation	Infrastructure requirements; regulatory approval pending
DEEP-Lung IV	NCT04994795, Ref. [56]	Clinical + pathological + baseline CT radiomics	>4000 NSCLC patients (first-line ICI)	AUC 0.85 (proof of concept, n = 63)	Real-world multicentric design; all modalities contribute to prediction	Proof of concept phase; requires full cohort validation

**Table 3:** Multimodal Biomarker Integration Studies for Immunotherapy Prediction in NSCLC.

Study/Reference	Modalities Integrated	AI/ML Method	Outcome Predicted	Performance	Comparator (Unimodal)	Key Insight
Ref. [57]	ctDNA genomic features (25 prognostic features from pre-treatment profiling)	XGBoost (gradient boosting)	PFS in NSCLC patients receiving ICIs	AUC: 0.82 (training), 0.79 (validation), 0.77 (test)	Not reported	Identified TP53, BRCA2, and NOTCH1 as key predictive biomarkers from ctDNA
Refs. [58,59,60]	Clinical + genomic data + histology radiomics + deep features	Deep learning models	Treatment response	AUC 0.80–0.90	Unimodal approaches (lower AUC)	Multimodal deep learning consistently outperforms single-modality models
Ref. [61]	Clinical features (modified lung immune predictive index) + radiomics score + immune signatures	Multi-dimensional predictive model	PFS and OS	C-index: 0.721 (PFS), 0.727 (OS)	Individual components (lower C-index)	Combined clinical-radiomic-immune model superior to any single component
Ref. [62]	Radiomics + pathological biomarkers (PD-L1, STK11, KRAS) + clinical variables	Multiomic graph model	PFS prediction	C-index: 0.71 (95% CI 0.61–0.72)	Combination clinical: C-index 0.68; Clinical-only: C-index 0.58	Multiomic integration outperforms clinical and combination clinical models
Ref. [63]	Inflammatory indices (NLR, PLR) + PD-L1 expression + molecular alterations	Combined clustering model	ORR and patient stratification	Identified clusters with ORR ranging 7–41%	Single biomarker stratification	Inflammatory-molecular clusters identify distinct prognostic patient subgroups
Ref. [55] (LC-NICER)	Longitudinal radiomics + deep learning TME + habitat imaging	Multimodal deep learning	Pathologic response to neoadjuvant IO-chemo	10% absolute improvement over PD-L1; 18% over RECIST 1.1	PD-L1 IHC and RECIST 1.1	Prospective multicenter validation of integrated radiomic-TME model
Ref. [56] (DEEP-Lung IV)	Clinical + pathological + baseline CT radiomics	Logistic regression (proof of concept); deep learning (full study)	Response at first evaluation	AUC 0.85 (n = 63, proof of concept)	Not reported	All data modalities contributed to model performance in real-world multicentric setting

## Data Availability

No new data was created or analyzed in this study.

## Abbreviations

AUROC, area under the receiver operating characteristic curve; AUC, area under the curve; C-index, concordance index; CNN, convolutional neural network; CT, computed tomography; ctDNA, circulating tumor DNA; GNN, graph neural network; ICI, immune checkpoint inhibitor; IHC, immunohistochemistry; MRD, molecular residual disease; NLR, neutrophil-to-lymphocyte ratio; NSCLC, non-small cell lung cancer; ORR, objective response rate; OS, overall survival; PD-L1, programmed death ligand-1; PET, positron emission tomography; PFS, progression-free survival; PLR, platelet-to-lymphocyte ratio; RE-ViT, Radiomics-Embedded Vision Transformer; RECIST, Response Evaluation Criteria in Solid Tumors; TME, tumor microenvironment; ViT, Vision Transformer.
